# {5-Chloro-2-[(2-hy­droxy­benzyl­idene)amino]­phen­yl}(phen­yl)methanone

**DOI:** 10.1107/S1600536811048690

**Published:** 2011-11-25

**Authors:** M. Aslam, I. Anis, N. Afza, A. Nelofar, S. Yousuf

**Affiliations:** a Pharmaceutical Research Centre, PCSIR Laboratories Complex, Karachi, Pakistan; b Department of Chemistry, University of Karachi, Karachi, Pakistan; c H.E.J. Research Institute of Chemistry, International Center for Chemical and Biological Sciences, University of Karachi, Karachi 75270, Pakistan

## Abstract

The title Schiff base compound, C_20_H_14_ClNO_2_, adopts an *E* configuration about the azomethine bond. The phenol and chloro­benzene rings form dihedral angles of 84.71 (9) and 80.70 (8)°, respectively, with the phenyl ring and are twisted by 15.32 (8)° with respect to one another. The mol­ecular conformation is stabilized by an intra­molecular O—H⋯N hydrogen bond, which forms an *S*(6) ring motif. In the crystal, mol­ecules are linked by C—H⋯O hydrogen bonds, forming columns parallel to the *a* axis.

## Related literature

For the biological activity of Schiff bases, see: Khan *et al.* (2009 ▶); Gerdemann *et al.* (2002 ▶); Samadhiya & Halve (2001 ▶); Mallikarjun & Sangamesh (1997 ▶); Fioravanti *et al.* (1995 ▶); Solomon & Lowery (1993 ▶). For related structures, see: Aslam *et al.* (2011 ▶); Zeb & Yousuf (2011 ▶); Cox *et al.* (2008 ▶); Vasco-Mendez *et al.* (1996 ▶).
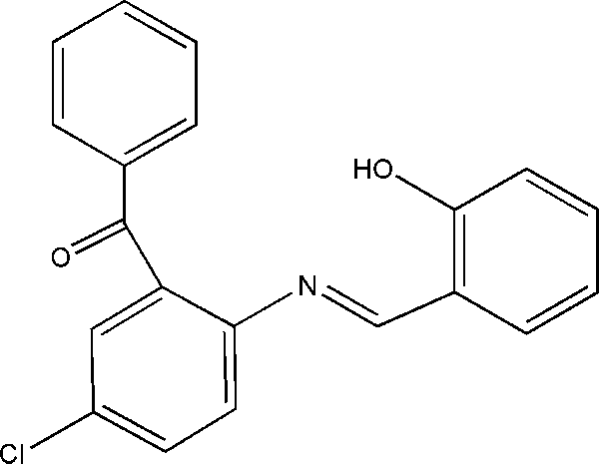


## Experimental

### 

#### Crystal data

C_20_H_14_ClNO_2_
*M* *_r_* = 335.77
Triclinic, 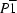

*a* = 7.3904 (9) Å
*b* = 10.7933 (14) Å
*c* = 10.8999 (14) Å
α = 73.120 (2)°
β = 87.919 (3)°
γ = 82.953 (3)°
*V* = 825.71 (18) Å^3^
*Z* = 2
Mo *K*α radiation
μ = 0.24 mm^−1^
*T* = 273 K
0.43 × 0.19 × 0.16 mm

#### Data collection

Bruker SMART APEX CCD area-detector diffractometer
Absorption correction: multi-scan (*SADABS*; Bruker, 2000 ▶) *T*_min_ = 0.903, *T*_max_ = 0.962
9338 measured reflections
3066 independent reflections
2481 reflections with *I* > 2σ(*I*)
*R*_int_ = 0.016

#### Refinement

*R*[*F* ^2^ > 2σ(*F* ^2^)] = 0.038
*wR*(*F* ^2^) = 0.109
*S* = 1.03
3066 reflections
221 parameters
1 restraint
H atoms treated by a mixture of independent and constrained refinement
Δρ_max_ = 0.17 e Å^−3^
Δρ_min_ = −0.20 e Å^−3^

### 

Data collection: *SMART* (Bruker, 2000 ▶); cell refinement: *SAINT* (Bruker, 2000 ▶); data reduction: *SAINT*; program(s) used to solve structure: *SHELXS97* (Sheldrick, 2008 ▶); program(s) used to refine structure: *SHELXL97* (Sheldrick, 2008 ▶); molecular graphics: *SHELXTL* (Sheldrick, 2008 ▶); software used to prepare material for publication: *SHELXTL*, *PARST* (Nardelli, 1995 ▶) and *PLATON* (Spek, 2009 ▶).

## Supplementary Material

Crystal structure: contains datablock(s) global, I. DOI: 10.1107/S1600536811048690/rz2667sup1.cif

Structure factors: contains datablock(s) I. DOI: 10.1107/S1600536811048690/rz2667Isup2.hkl

Supplementary material file. DOI: 10.1107/S1600536811048690/rz2667Isup3.cml

Additional supplementary materials:  crystallographic information; 3D view; checkCIF report

## Figures and Tables

**Table 1:** Hydrogen-bond geometry (Å, °)

*D*—H⋯*A*	*D*—H	H⋯*A*	*D*⋯*A*	*D*—H⋯*A*
O2—H2*A*⋯N1	0.89 (2)	1.80 (2)	2.6188 (19)	152 (2)
C7—H7*A*⋯O1^i^	0.93	2.57	3.353 (2)	142
C17—H17*A*⋯O1^ii^	0.93	2.48	3.340 (2)	155

Symmetry codes: (i) 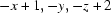; (ii) 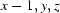.

**Fig. 1.**
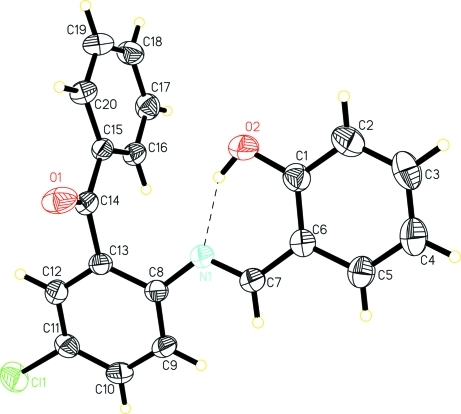
The molecular structure of the title compound with displacement ellipsoids drawn at the 30% probability level.

**Fig. 2.**
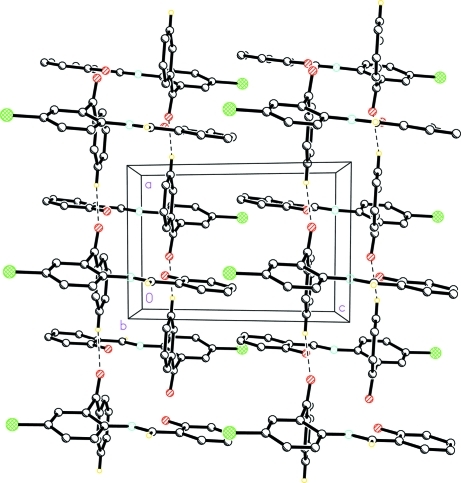
The crystal packing of the title compound viewed along the b axis. Hydrogen atoms not involved in hydrogen bonding (dashed lines) are omitted.

## supplementary crystallographic information

### Comment

Schiff bases are well known ligands in coordination chemistry with a wide range of biological activities (Khan *et al.*, 2009; Gerdemann *et al.*, 2002; Samadhiya & Halve, 2001; Mallikarjun & Sangamesh, 1997; Fioravanti *et al.*, 1995; Solomon & Lowery, 1993). The title compound was prepared as a part of our ongoing research on bioactive compounds.

The structure of title compound (Fig. 1) is similar to that of the recently reported compound {5-chloro-2-[(4-nitrobenzylidene)amino]phenyl}(phenyl)methanone (Aslam *et al.*, 2011) with the difference that the nitrobenzene moiety is replaced by a phenol group. The phenol (C1-C6) and clorobenzene (C8-C13) rings are twisted by 15.32 (8)° and form dihedral angles of 84.71 (9)° and 80.70 (8)°, respectively, with the phenyl ring (C15-C20). The azomethine C═N double bond adopts an *E* configuration, with the C8/N1/C6-C7 torsion angle of 178.60 (13)°. The molecular conformation is stabilized by an O2—H2A···N1 intramolecular hydrogen bond (Table 1) to form a *S*6 ring motif (Fig. 1). Bond lengths and angles are similar to those observed in other structurally related compounds (Aslam *et al.*, 2011; Cox *et al.*, 2008; Vasco-Mendez *et al.*, 1996; Zeb & Yousuf, 2011). In the crystal structure, the molecules are linked to form columns parallel to the *a* axis (Fig. 2) *via* C7—H7A···O1 and C17—H17A···O1 intermolecular hydrogen bonds (Table 1).

### Experimental

The synthesis of title compound was carried out by refluxing a mixture of salicylaldehyde (1 mol) and 2-amino-5-chlorobenzophenone (1 mol) in ethanol (50 ml) along with 3 drops of conc. H_2_SO_4_ for 5 h at 343 K. After cooling the mixture was concentrated to one third under reduced pressure followed by addition of ethyl acetate (10 ml) and chloroforom (10 ml). The mixture was kept at room temperature and yellow crystals were obtained after seven days. The crystalline product was collected, washed with methanol and dried to afford the title compound in 85% yield. Slow evaporation of a methanol solution afforded yellow crystals found suitable for single-crystal X-ray diffraction studies. All chemicals were purchased from Sigma-Aldrich.

### Refinement

All C-bound H atoms were positioned geometrically with C—H = 0.93 Å, and constrained to ride on their parent atoms with *U*_iso_(H)= 1.2*U*_eq_(C). The hydroxy H atom atom was located in a difference Fourier map and refined isotropically. During the refinement, the C1···H2A separation was constrained to be 1.80 (2) Å.

### Crystal data

**Table:**

C_20_H_14_ClNO_2_	*Z* = 2
*M**_r_* = 335.77	*F*(000) = 348
Triclinic, *P*1	*D*_x_ = 1.351 Mg m^−^^3^
Hall symbol: -P 1	Mo *K*α radiation, λ = 0.71073 Å
*a* = 7.3904 (9) Å	Cell parameters from 3265 reflections
*b* = 10.7933 (14) Å	θ = 2.0–25.5°
*c* = 10.8999 (14) Å	µ = 0.24 mm^−^^1^
α = 73.120 (2)°	*T* = 273 K
β = 87.919 (3)°	Block, yellow
γ = 82.953 (3)°	0.43 × 0.19 × 0.16 mm
*V* = 825.71 (18) Å^3^	

### Data collection

**Table:**

Bruker SMART APEX CCD area-detector diffractometer	3066 independent reflections
Radiation source: fine-focus sealed tube	2481 reflections with *I* > 2σ(*I*)
graphite	*R*_int_ = 0.016
ω scan	θ_max_ = 25.5°, θ_min_ = 2.0°
Absorption correction: multi-scan (*SADABS*; Bruker, 2000)	*h* = −8→8
*T*_min_ = 0.903, *T*_max_ = 0.962	*k* = −13→13
9338 measured reflections	*l* = −13→13

### Refinement

**Table:**

Refinement on *F*^2^	Primary atom site location: structure-invariant direct methods
Least-squares matrix: full	Secondary atom site location: difference Fourier map
*R*[*F*^2^ > 2σ(*F*^2^)] = 0.038	Hydrogen site location: inferred from neighbouring sites
*wR*(*F*^2^) = 0.109	H atoms treated by a mixture of independent and constrained refinement
*S* = 1.03	*w* = 1/[σ^2^(*F*_o_^2^) + (0.0541*P*)^2^ + 0.1538*P*] where *P* = (*F*_o_^2^ + 2*F*_c_^2^)/3
3066 reflections	(Δ/σ)_max_ < 0.001
221 parameters	Δρ_max_ = 0.17 e Å^−^^3^
1 restraint	Δρ_min_ = −0.20 e Å^−^^3^

### Special details

**Table:**

Geometry. All e.s.d.'s (except the e.s.d. in the dihedral angle between two l.s. planes) are estimated using the full covariance matrix. The cell e.s.d.'s are taken into account individually in the estimation of e.s.d.'s in distances, angles and torsion angles; correlations between e.s.d.'s in cell parameters are only used when they are defined by crystal symmetry. An approximate (isotropic) treatment of cell e.s.d.'s is used for estimating e.s.d.'s involving l.s. planes.
Refinement. Refinement of *F*^2^ against ALL reflections. The weighted *R*-factor *wR* and goodness of fit *S* are based on *F*^2^, conventional *R*-factors *R* are based on *F*, with *F* set to zero for negative *F*^2^. The threshold expression of *F*^2^ > σ(*F*^2^) is used only for calculating *R*-factors(gt) *etc*. and is not relevant to the choice of reflections for refinement. *R*-factors based on *F*^2^ are statistically about twice as large as those based on *F*, and *R*- factors based on ALL data will be even larger.

### Fractional atomic coordinates and isotropic or equivalent isotropic displacement parameters (Å^2^)

**Table:**

	*x*	*y*	*z*	*U*_iso_*/*U*_eq_	
Cl1	0.31204 (8)	0.24660 (5)	0.46997 (5)	0.0854 (2)	
O1	0.59969 (16)	−0.21801 (12)	0.84282 (15)	0.0757 (4)	
O2	0.2754 (2)	−0.32066 (12)	1.15776 (14)	0.0788 (4)	
H2A	0.279 (3)	−0.254 (2)	1.0879 (11)	0.103 (8)*	
N1	0.26996 (17)	−0.08203 (12)	1.00695 (12)	0.0487 (3)	
C1	0.2475 (2)	−0.26589 (17)	1.25483 (17)	0.0612 (4)	
C2	0.2341 (3)	−0.3472 (2)	1.3787 (2)	0.0828 (6)	
H2B	0.2496	−0.4372	1.3938	0.099*	
C3	0.1979 (3)	−0.2949 (3)	1.4788 (2)	0.0882 (7)	
H3A	0.1878	−0.3502	1.5613	0.106*	
C4	0.1761 (3)	−0.1621 (3)	1.45934 (19)	0.0817 (6)	
H4A	0.1495	−0.1278	1.5279	0.098*	
C5	0.1939 (2)	−0.0807 (2)	1.33794 (17)	0.0657 (5)	
H5A	0.1819	0.0089	1.3249	0.079*	
C6	0.2299 (2)	−0.13059 (16)	1.23310 (15)	0.0519 (4)	
C7	0.2450 (2)	−0.04203 (15)	1.10631 (15)	0.0490 (4)	
H7A	0.2363	0.0470	1.0967	0.059*	
C8	0.28094 (19)	0.00455 (14)	0.88212 (14)	0.0454 (3)	
C9	0.2189 (2)	0.13726 (15)	0.84612 (16)	0.0541 (4)	
H9A	0.1692	0.1759	0.9076	0.065*	
C10	0.2302 (2)	0.21192 (15)	0.72062 (17)	0.0580 (4)	
H10A	0.1894	0.3006	0.6976	0.070*	
C11	0.3023 (2)	0.15451 (16)	0.62954 (16)	0.0557 (4)	
C12	0.3654 (2)	0.02359 (16)	0.66243 (16)	0.0550 (4)	
H12A	0.4144	−0.0142	0.6001	0.066*	
C13	0.3554 (2)	−0.05126 (14)	0.78849 (15)	0.0463 (4)	
C14	0.4369 (2)	−0.19231 (15)	0.82362 (15)	0.0487 (4)	
C15	0.3180 (2)	−0.29461 (14)	0.83004 (14)	0.0449 (3)	
C16	0.1298 (2)	−0.26661 (16)	0.81996 (15)	0.0529 (4)	
H16A	0.0760	−0.1813	0.8075	0.063*	
C17	0.0220 (2)	−0.36421 (18)	0.82830 (17)	0.0626 (5)	
H17A	−0.1041	−0.3450	0.8220	0.075*	
C18	0.1019 (3)	−0.49073 (17)	0.84612 (18)	0.0657 (5)	
H18A	0.0294	−0.5568	0.8520	0.079*	
C19	0.2881 (3)	−0.51924 (16)	0.85517 (19)	0.0672 (5)	
H19A	0.3412	−0.6045	0.8667	0.081*	
C20	0.3961 (2)	−0.42217 (15)	0.84726 (16)	0.0558 (4)	
H20A	0.5221	−0.4421	0.8535	0.067*	

### Atomic displacement parameters (Å^2^)

**Table:**

	*U*^11^	*U*^22^	*U*^33^	*U*^12^	*U*^13^	*U*^23^
Cl1	0.1056 (4)	0.0725 (3)	0.0594 (3)	−0.0004 (3)	0.0066 (3)	0.0054 (2)
O1	0.0506 (7)	0.0541 (7)	0.1234 (12)	0.0019 (5)	−0.0083 (7)	−0.0295 (7)
O2	0.1159 (12)	0.0473 (7)	0.0672 (9)	−0.0021 (7)	0.0064 (8)	−0.0111 (7)
N1	0.0531 (7)	0.0435 (7)	0.0492 (8)	−0.0035 (6)	0.0016 (6)	−0.0137 (6)
C1	0.0636 (11)	0.0598 (10)	0.0556 (10)	−0.0029 (8)	−0.0010 (8)	−0.0113 (8)
C2	0.0990 (16)	0.0672 (12)	0.0684 (13)	−0.0047 (11)	−0.0030 (11)	0.0004 (10)
C3	0.0985 (16)	0.1036 (18)	0.0499 (11)	−0.0121 (13)	−0.0008 (10)	−0.0024 (11)
C4	0.0866 (14)	0.1068 (18)	0.0524 (11)	−0.0116 (12)	0.0000 (10)	−0.0237 (11)
C5	0.0666 (11)	0.0776 (12)	0.0561 (11)	−0.0078 (9)	−0.0022 (8)	−0.0241 (9)
C6	0.0455 (8)	0.0593 (10)	0.0506 (9)	−0.0053 (7)	−0.0025 (7)	−0.0152 (7)
C7	0.0470 (8)	0.0467 (8)	0.0546 (9)	−0.0046 (6)	−0.0012 (7)	−0.0171 (7)
C8	0.0447 (8)	0.0415 (8)	0.0506 (9)	−0.0056 (6)	0.0003 (6)	−0.0139 (7)
C9	0.0634 (10)	0.0412 (8)	0.0596 (10)	−0.0012 (7)	0.0017 (8)	−0.0196 (7)
C10	0.0666 (10)	0.0385 (8)	0.0650 (11)	−0.0022 (7)	−0.0036 (8)	−0.0103 (8)
C11	0.0580 (10)	0.0499 (9)	0.0536 (9)	−0.0073 (7)	0.0001 (7)	−0.0058 (7)
C12	0.0596 (10)	0.0524 (9)	0.0520 (9)	−0.0025 (7)	0.0058 (7)	−0.0160 (7)
C13	0.0451 (8)	0.0407 (8)	0.0533 (9)	−0.0054 (6)	0.0017 (6)	−0.0143 (7)
C14	0.0503 (9)	0.0450 (8)	0.0507 (9)	−0.0005 (7)	0.0033 (7)	−0.0159 (7)
C15	0.0519 (8)	0.0416 (8)	0.0401 (8)	−0.0008 (6)	0.0008 (6)	−0.0122 (6)
C16	0.0542 (9)	0.0477 (9)	0.0551 (9)	0.0013 (7)	0.0013 (7)	−0.0153 (7)
C17	0.0517 (9)	0.0691 (11)	0.0684 (11)	−0.0096 (8)	0.0044 (8)	−0.0217 (9)
C18	0.0742 (12)	0.0551 (10)	0.0701 (12)	−0.0202 (9)	0.0031 (9)	−0.0167 (9)
C19	0.0777 (13)	0.0423 (9)	0.0805 (13)	−0.0027 (8)	−0.0081 (10)	−0.0169 (8)
C20	0.0565 (9)	0.0457 (9)	0.0640 (10)	0.0024 (7)	−0.0046 (8)	−0.0167 (8)

### Geometric parameters (Å, °)

**Table:**

Cl1—C11	1.7386 (17)	C9—C10	1.377 (2)
O1—C14	1.2125 (18)	C9—H9A	0.9300
O2—C1	1.352 (2)	C10—C11	1.375 (2)
O2—H2A	0.882 (19)	C10—H10A	0.9300
N1—C7	1.277 (2)	C11—C12	1.378 (2)
N1—C8	1.4154 (19)	C12—C13	1.382 (2)
C1—C2	1.388 (3)	C12—H12A	0.9300
C1—C6	1.400 (2)	C13—C14	1.510 (2)
C2—C3	1.371 (3)	C14—C15	1.479 (2)
C2—H2B	0.9300	C15—C16	1.388 (2)
C3—C4	1.377 (3)	C15—C20	1.388 (2)
C3—H3A	0.9300	C16—C17	1.377 (2)
C4—C5	1.371 (3)	C16—H16A	0.9300
C4—H4A	0.9300	C17—C18	1.381 (3)
C5—C6	1.402 (2)	C17—H17A	0.9300
C5—H5A	0.9300	C18—C19	1.373 (3)
C6—C7	1.444 (2)	C18—H18A	0.9300
C7—H7A	0.9300	C19—C20	1.375 (2)
C8—C9	1.393 (2)	C19—H19A	0.9300
C8—C13	1.393 (2)	C20—H20A	0.9300
			
C1—O2—H2A	105.0 (12)	C9—C10—H10A	120.2
C7—N1—C8	122.32 (13)	C10—C11—C12	120.90 (15)
O2—C1—C2	118.41 (17)	C10—C11—Cl1	120.10 (13)
O2—C1—C6	121.81 (15)	C12—C11—Cl1	119.00 (14)
C2—C1—C6	119.78 (18)	C11—C12—C13	119.61 (15)
C3—C2—C1	120.0 (2)	C11—C12—H12A	120.2
C3—C2—H2B	120.0	C13—C12—H12A	120.2
C1—C2—H2B	120.0	C12—C13—C8	120.47 (14)
C2—C3—C4	121.19 (19)	C12—C13—C14	118.56 (14)
C2—C3—H3A	119.4	C8—C13—C14	120.87 (13)
C4—C3—H3A	119.4	O1—C14—C15	121.85 (14)
C5—C4—C3	119.4 (2)	O1—C14—C13	118.73 (14)
C5—C4—H4A	120.3	C15—C14—C13	119.39 (13)
C3—C4—H4A	120.3	C16—C15—C20	119.02 (14)
C4—C5—C6	121.1 (2)	C16—C15—C14	121.71 (13)
C4—C5—H5A	119.5	C20—C15—C14	119.26 (14)
C6—C5—H5A	119.5	C17—C16—C15	120.48 (15)
C1—C6—C5	118.54 (16)	C17—C16—H16A	119.8
C1—C6—C7	121.87 (15)	C15—C16—H16A	119.8
C5—C6—C7	119.59 (16)	C16—C17—C18	119.74 (16)
N1—C7—C6	122.09 (15)	C16—C17—H17A	120.1
N1—C7—H7A	119.0	C18—C17—H17A	120.1
C6—C7—H7A	119.0	C19—C18—C17	120.21 (16)
C9—C8—C13	118.64 (14)	C19—C18—H18A	119.9
C9—C8—N1	125.53 (14)	C17—C18—H18A	119.9
C13—C8—N1	115.80 (13)	C18—C19—C20	120.23 (16)
C10—C9—C8	120.82 (15)	C18—C19—H19A	119.9
C10—C9—H9A	119.6	C20—C19—H19A	119.9
C8—C9—H9A	119.6	C19—C20—C15	120.31 (16)
C11—C10—C9	119.56 (15)	C19—C20—H20A	119.8
C11—C10—H10A	120.2	C15—C20—H20A	119.8
			
O2—C1—C2—C3	−177.1 (2)	C11—C12—C13—C8	0.4 (2)
C6—C1—C2—C3	2.1 (3)	C11—C12—C13—C14	−175.92 (15)
C1—C2—C3—C4	−0.7 (4)	C9—C8—C13—C12	−0.7 (2)
C2—C3—C4—C5	−1.1 (4)	N1—C8—C13—C12	177.30 (13)
C3—C4—C5—C6	1.4 (3)	C9—C8—C13—C14	175.60 (14)
O2—C1—C6—C5	177.45 (16)	N1—C8—C13—C14	−6.4 (2)
C2—C1—C6—C5	−1.7 (3)	C12—C13—C14—O1	82.0 (2)
O2—C1—C6—C7	−1.6 (3)	C8—C13—C14—O1	−94.33 (19)
C2—C1—C6—C7	179.24 (16)	C12—C13—C14—C15	−96.24 (17)
C4—C5—C6—C1	0.0 (3)	C8—C13—C14—C15	87.42 (18)
C4—C5—C6—C7	179.05 (16)	O1—C14—C15—C16	173.85 (16)
C8—N1—C7—C6	178.60 (13)	C13—C14—C15—C16	−8.0 (2)
C1—C6—C7—N1	2.1 (2)	O1—C14—C15—C20	−5.8 (2)
C5—C6—C7—N1	−176.98 (15)	C13—C14—C15—C20	172.40 (14)
C7—N1—C8—C9	−18.6 (2)	C20—C15—C16—C17	0.7 (2)
C7—N1—C8—C13	163.59 (14)	C14—C15—C16—C17	−178.96 (15)
C13—C8—C9—C10	0.2 (2)	C15—C16—C17—C18	−0.4 (3)
N1—C8—C9—C10	−177.59 (15)	C16—C17—C18—C19	−0.2 (3)
C8—C9—C10—C11	0.6 (3)	C17—C18—C19—C20	0.4 (3)
C9—C10—C11—C12	−0.8 (3)	C18—C19—C20—C15	0.0 (3)
C9—C10—C11—Cl1	178.32 (13)	C16—C15—C20—C19	−0.5 (2)
C10—C11—C12—C13	0.3 (3)	C14—C15—C20—C19	179.16 (16)
Cl1—C11—C12—C13	−178.83 (12)		

### Hydrogen-bond geometry (Å, °)

**Table:**

*D*—H···*A*	*D*—H	H···*A*	*D*···*A*	*D*—H···*A*
O2—H2A···N1	0.89 (2)	1.80 (2)	2.6188 (19)	152.(2)
C7—H7A···O1^i^	0.93	2.57	3.353 (2)	142.
C17—H17A···O1^ii^	0.93	2.48	3.340 (2)	155.

Symmetry codes: (i) −*x*+1, −*y*, −*z*+2; (ii) *x*−1, *y*, *z*.
